# Infectious Agents in Atherosclerotic Cardiovascular Diseases through Oxidative Stress

**DOI:** 10.3390/ijms18112459

**Published:** 2017-11-18

**Authors:** Marisa Di Pietro, Simone Filardo, Francesca Falasca, Ombretta Turriziani, Rosa Sessa

**Affiliations:** 1 Department of Public Health and Infectious Diseases, Sapienza University, Piazzale Aldo Moro, 5, 00185 Rome, Italy; marisa.dipietro@uniroma1.it (M.D.P.); simone.filardo@uniroma1.it (S.F.); 2 Department of Molecular Medicine, Sapienza University, Viale di Porta Tiburtina, 28, 00185 Rome, Italy; francesca.falasca@uniroma1.it (F.F.); ombretta.turriziani@uniroma1.it (O.T.)

**Keywords:** infectious agents, atherosclerotic cardiovascular diseases, oxidative stress

## Abstract

Accumulating evidence demonstrates that vascular oxidative stress is a critical feature of atherosclerotic process, potentially triggered by several infectious agents that are considered as risk co-factors for the atherosclerotic cardiovascular diseases (CVDs). *C. pneumoniae* has been shown to upregulate multiple enzymatic systems capable of producing reactive oxygen species (ROS) such as NADPH oxidase (NOX) and cyclooxygenase in vascular endothelial cells, NOX and cytochrome c oxidase in macrophages as well as nitric oxide synthase and lipoxygenase in platelets contributing to both early and late stages of atherosclerosis. *P. gingivalis* seems to be markedly involved in the atherosclerotic process as compared to *A. actinomycetemcomitans* contributing to LDL oxidation and foam cell formation. Particularly interesting is the evidence describing the NLRP3 inflammasome activation as a new molecular mechanism underlying *P. gingivalis*-induced oxidative stress and inflammation. Amongst viral agents, immunodeficiency virus-1 and hepatitis C virus seem to have a major role in promoting ROS production, contributing, hence, to the early stages of atherosclerosis including endothelial dysfunction and LDL oxidation. In conclusion, oxidative mechanisms activated by several infectious agents during the atherosclerotic process underlying CVDs are very complex and not well-known, remaining, thus, an attractive target for future research.

## 1. Introduction

Atherosclerotic cardiovascular diseases (CVDs) continue to represent a major public health problem in developed countries, accounting for one-third of all deaths worldwide [1]. The underlying pathological process is the atherosclerosis, a slowly progressing chronic inflammatory disorder of the arterial wall, characterized by the formation, progression, and destabilization of atherosclerotic plaque responsible for acute syndromes such as myocardial infarction and stroke.

Atherosclerosis typically begins with endothelial dysfunction, characterized by increased production of superoxide anion and by reduced nitric oxide bioavailability, and followed by low-density lipoprotein (LDL) oxidation and accumulation in macrophages with foam cell formation. Consequently, several pro-atherogenic mechanisms are activated, including leukocyte adhesion and migration, vascular smooth muscle cell (VSMC) proliferation, as well as platelet adhesion and aggregation, leading to an unstable atherosclerotic plaque [2].

Several risk factors for atherosclerosis have been identified, including traditional factors such as hypertension, diabetes, dyslipidemia, and smoking, and non-traditional risk factors including inflammation and oxidative stress.

Oxidative stress, resulting from the imbalance between the production of reactive oxygen species (ROS) and the activity of antioxidant systems, has recently acquired increasing importance. Indeed, oxidative stress has been shown to play a key role in all stages of the atherosclerotic process from the formation to the rupture of vascular plaque [3,4].

In the vascular wall, macrophages, endothelial cells, and VSMCs have enzymatic systems capable of producing ROS such as nicotinamide adenine dinucleotide phosphate (NADPH) oxidase (NOX), xanthine oxidase (XO), uncoupled endothelial nitric oxide synthase, and mitochondrial electron transport chain as well as antioxidant enzyme systems that detoxify ROS, including superoxide dismutases (SOD), catalase, glutathione peroxidases (GPx), and paraoxonases [4,5].

ROS, at moderate concentrations, act as signaling molecules and are essential in the regulation of vascular tone, cell growth and proliferation, and apoptosis, whereas ROS overproduction leads to oxidative stress, promoting atherogenesis [3,5].

In addition to oxidative stress, another central underlying driver of the atherosclerotic process is the inflammation that promotes the initiation and the evolution of atheroma contributing to the precipitation of acute thrombotic complications of unstable plaque [6,7].

Oxidative stress and inflammation play an interconnected and mutually reinforcing role to accelerate plaque formation and progression. Specifically, following the LDL oxidation, an increased production of adhesion molecules on the vascular surface occurs, resulting in the migration and infiltration of inflammatory cells into the vascular wall. Monocytes, lymphocytes, and mast cells, in turn, produce ROS, chemokines, interleukins, and proteases, increasing the risk of CVDs [8,9].

**Figure 1:**
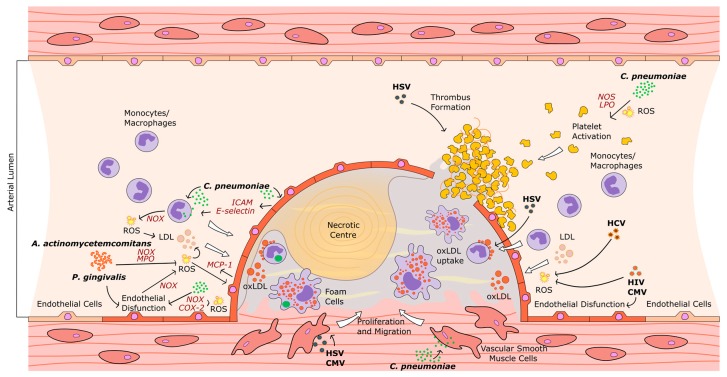
Putative pathways of infectious agent-mediated oxidative stress in several stages of the atherosclerotic process. *C. pneumoniae*, HIV, HCV, HSV, and CMV contribute to endothelial dysfunction; *P. gingivalis*, *A. actinomycetemcomitans*, *C. pneumoniae*, HIV, HCV, and CMV trigger LDL oxidation; *P. gingivalis*, *C. pneumoniae*, and CMV induce macrophage-derived foam cell formation; *P. gingivalis*, *C. pneumoniae*, HSV, and CMV contribute to progression of atherosclerotic plaque (ICAM: intercellular adhesion molecule; NOX: nicotinamide adenine dinucleotide phosphate oxidase; MPO: myeloperoxidase; NOS: nitric oxide synthase; COX-2: cyclooxygenase-2; LPO: lipoxygenase).

Currently, there is increasing evidence that vascular oxidative stress as well as inflammation may be triggered by infectious agents such as periodontal pathogens (*Porphyromonas gingivalis* and *Aggregatibacter actinomycetemcomitans)*, *Chlamydia pneumoniae*, immunodeficiency virus-1 (HIV), hepatitis C virus (HCV), herpes simplex virus type 2 (HSV-2), and cytomegalovirus (CMV), considered as risk co-factors for CVDs (Figure 1) [10,11,12,13,14,15,16,17].

In this review, we focus on the oxidative mechanisms through which infectious agents may contribute to the early stages of the atherosclerotic process, by promoting endothelial dysfunction, LDL oxidation, and foam cell formation, and to the late stages, by stimulating platelet activation and VSMC migration and proliferation. Furthermore, potential strategies, targeting pathogen-induced oxidative stress in the prevention of CVDs, are also discussed.

## 2. Bacterial Infectious Agents

### 2.1. Periodontal Pathogens

Amongst the numerous periodontal pathogens involved in the onset and/or development of CVDs, *P. gingivalis* and *A. actinomycetemcomitans* are the sole viable bacteria detected in atherosclerotic lesions [16]. Considerable evidence indicates that *P. gingivalis* and *A. actinomycetemcomitans* can disseminate in the bloodstream causing bacteremia and localize in the vascular wall contributing to the atherosclerotic process by inducing ROS-mediated LDL oxidation [14].

Concerning *A. actinomycetemcomitans*, ROS production has been demonstrated to depend on NOX and myeloperoxidase activities in apolipoprotein E-deficient spontaneously hyperlipidemic mice (14); on the contrary, *P. gingivalis*-induced ROS generation depended on the interaction between bacterial gingipain R and platelets and/or between platelets and neutrophils [18,19].

*P. gingivalis* has also been shown to promote the foam cell formation by augmenting the uptake of oxidized LDL (ox-LDL) into macrophages through the activation of scavenger receptor (cluster of differentiation 36) [20], as well as to contribute to the progression of atherosclerotic plaque by stimulating the release in the vascular environment of monocyte chemoattractant protein-1 (MCP-1). Specifically, the induction of MCP-1 in vascular endothelial cells in response to *P. gingivalis* appears to be dependent on NOX-mediated superoxide anion production followed by the activation of multiple signaling pathways (p38 mitogen-activated protein, kinase, c-Jun N-terminal kinase, nuclear factor-κB, and activator protein-1) [21].

Over the last few years, studies have proposed a new mechanism by which *P. gingivalis* may promote the progression of atherosclerotic plaque. In particular, the ability of *P. gingivalis* to induce endothelial cell apoptosis in the presence of ox-LDL leading to the formation of necrotic core has been evidenced [22].

Furthermore, there is evidence that *P. gingivalis* may also contribute to cardiac rupture by enhancing matrix metalloproteinases-9 activity through impairing autophagy-mediated clearance of damaged mitochondria, and, hence, increasing oxidative stress [23].

Particularly important are recent experimental studies that highlighted the molecular mechanisms linking oxidative stress to inflammation in *P. gingivalis*-mediated atherosclerosis. In particular, *P. gingivalis*-induced ROS production has been shown to activate the NOD-like receptor family, the NLR pyrin domain containing 3 (NLRP3) inflammasome in macrophages, leading to an increased production of atherogenic cytokines such as interleukin (IL)-1β, IL-18, and tumor necrosis factor-α (TNF-α). [24].

### 2.2. Chlamydia pneumoniae

*C. pneumoniae,* a common cause of respiratory infections, is considered as the most plausible additional risk factor for CVDs due to the highest number of experimental studies to date demonstrating an association with atherosclerosis [25,26,27]. Importantly, *C. pneumoniae* has been shown to disseminate via the bloodstream to the vascular wall as evidenced by the presence of chlamydial DNA in peripheral blood mononuclear cells as well as in atherosclerotic lesions [28,29,30].

Even more important are the isolation of viable bacteria from the atheroma and the atherogenic effects of *C. pneumoniae* observed on the vascular wall [27]. Indeed, studies have demonstrated that *C. pneumoniae* is also able to induce oxidative stress and, hence, contribute to the early stages of the atherosclerotic process by promoting endothelial dysfunction, LDL oxidation, and foam cell formation, and to the late stages, by stimulating platelet activation [31].

Regarding endothelial dysfunction, *C. pneumoniae* has been shown to significantly enhance the superoxide anion production in endothelial cells through the upregulation of NOX (NOX-1, NOX-4, and p22phox), and cyclooxygenase-2 (COX-2) as well as through the downregulation of antioxidant enzyme systems such as catalase, SOD-1, and thioredoxin-1 [32].

In addition, there is also the evidence that *C. pneumoniae* may contribute to endothelial dysfunction by inactivating nitric oxide through ROS produced by VSMCs [33].

As for LDL oxidation, experimental studies have demonstrated the ability of *C. pneumoniae* to enhance the ROS production in both macrophages through NOX and cytochrome c oxidase activation and in platelets, through the activation of multiple enzymatic mechanisms, such as nitric oxide synthase and lipoxygenase [34,35,36].

Particularly interesting are recent in vitro studies, on 3D infection model of the intima, demonstrating that *C. pneumoniae*-mediated oxidative stress drives to different stages of atherosclerosis (recruitment of macrophages, LDL oxidation, foam cell formation, and endothelial dysfunction) [37].

Concerning the progression of atherosclerotic plaque, several potential mechanisms related to oxidative stress induced by *C. pneumoniae* have been suggested. First, both *C. pneumoniae* may upregulate the lectin-like ox-LDL receptor-1 (LOX-1), promoting the uptake ox-LDL in endothelial cells and, thereby, the formation of fatty streak. In particular, the activation of LOX-1 has been shown to depend on NOX and endothelial nitric oxide synthase activation [38].

Second, *C. pneumoniae* may enhance the expression of intercellular adhesion molecule 1 (ICAM) and E-selectin, known to play a critical role in the adhesion and migration of leukocytes into the vascular wall following the exposure to ox-LDL, exacerbating both oxidative stress as well as inflammation [39].

Third, in combination with ox-LDL, *C. pneumoniae* may augment ROS mediated-necrosis in macrophages as well as in endothelial cells, by accelerating the formation of atherosclerotic lipid-rich core and by worsening vascular inflammation [40,41].

Lastly, *C. pneumoniae* may contribute to platelet activation and, consequently, to thrombotic vascular occlusion during acute coronary events [42].

## 3. Viral Infectious Agents

### 3.1. HIV

Among HIV-positive patients, the prevalence of cardiovascular risk factors as well as the risk for cardiovascular events are higher than in HIV-negative individuals. In fact, several vascular complications, including coronary heart disease, pulmonary hypertension, and atherosclerosis, have been described in HIV-1-infected patients [43].

Endothelial dysfunction is a well-established response to cardiovascular risk factors, and it is considered a predictor of atherosclerosis. Endothelial dysfunction is characterized by the reduction of the bioavailability of vasodilators, such as nitric oxide, and/or the increase in endothelium-derived contracting factors [44], resulting in the impairment of endothelium-dependent vasodilation. In addition, endothelial dysfunction comprises also a specific state of endothelial activation, characterized by pro-inflammatory, proliferative, and pro-coagulatory conditions that favor atherogenesis.

In vitro studies have observed that HIV may promote endothelial dysfunction through its direct replication in endothelial cells [45], findings that are not supported by in vivo reports, since no replicating virus has ever been detected in endothelial cells, suggesting that a direct role of HIV in endothelial dysfunction is unlikely. However, increased HIV-RNA levels have been associated with endothelial dysfunction in HIV-positive patients [46]. In fact, several groups have reported an upregulation of cytokines and chemokines in HIV-positive patients, suggesting an indirect involvement of the virus by the means of pro-inflammatory cytokines production as well as oxidative stress induction [47]. The resulting inflammation, then, enhances the development of cardiovascular injury, atherosclerosis, and endothelial dysfunction [48].

The exact mechanism through which HIV-1 promotes oxidative stress remains largely unknown, even though the involvement of viral proteins, such as Tat and glycoprotein 120 (gp120), has been suggested. In fact, evidence show that the HIV-1 Tat and gp120 proteins promote ROS production and alter the regulation of antioxidant enzymes [12].

The Tat protein is a transcriptional activator of viral gene expression that infected cells produce early during the infection cycle and, then, release in the bloodstream, causing the transactivation of cellular genes. Specifically, Tat protein has been shown to increase ROS levels in cultured brain microvascular cells [49], to induce the activation of several ROS-producing enzymes [50], and to induce lipid peroxidation in rat endothelial cells [51]. Moreover, Tat protein is able to decrease the levels of antioxidants, including glutathione (GSH) [52], and to induce the expression of vascular cell adhesion molecule-1 (VCAM-1), triggering the ROS-mediated vascular inflammation [53]. Indeed, higher levels of cellular adhesion molecules have been observed in HIV-1 positive patients as compared to healthy donors [54]. It is known that in the early stages of atherosclerosis, adhesion molecules mediate leukocyte adhesion to the vascular endothelial cells participating in the formation of atherosclerotic plaque. Therefore, the Tat-mediated upregulation of cellular adhesion molecule gene and protein expression may represent a key molecular event in HIV-induced vascular injury.

The gp120 protein is usually exposed on the surface of HIV envelope and mediates the receptor binding. Several in vivo and in vitro studies suggested that this viral glycoprotein might have a double role in endothelial dysfunction, inducing either direct and/or indirect damage to the endothelium. Indeed, many studies have demonstrated the ability of gp120, virion-associated or -free, to promote apoptosis and induce the release of ET-1, further contributing to cellular disruption. In addition, gp120 may stimulate ROS release in numerous cell types as well as alter the regulation of antioxidant enzymes [12].

Although the introduction of combined antiretroviral therapy (cART) has dramatically reduced viral replication, HIV-1-infected patients treated with cART still showed a high risk of CVDs. In fact, like HIV, antiretroviral drugs seem able to increase ROS production and ROS-mediated effects. In particular, long-term exposure to nucleoside/nucleotide reverse transcriptase inhibitors (NRTIs) promotes endothelial ROS production, and d4T as well as AZT induce mitochondrial dysfunction and increase ROS generation [12]. In addition, a 6-month NRTIs treatment has been shown to reduce serum GSH levels in HIV-positive patients. Furthermore, HIV-1 protease inhibitors can cause the alteration of plasma lipoprotein metabolism (dyslipidemia) [55], contributing to endothelial dysfunction. Taken together, these findings show that HIV-1 as well as cART may have combined effects on the development and progression of vascular disorders through oxidative stress.

### 3.2. HCV

Hepatitis C virus (HCV) infection is a systemic disease that leads to increased risk of cirrhosis and its complications. In addition, HCV infection has been reported as a risk factor for subclinical and clinical cardiovascular diseases [56]. Preliminary studies on the general population showed that HCV infection was independently associated with atherosclerosis [57]. Later on, HCV RNA sequences were isolated within carotid plaques, suggesting, thus, that the direct replication of the virus within arterial walls may contribute to the onset of atherosclerosis and its complications [58]. In particular, HCV was shown to possess pro-atherogenic activity via the stimulation of pro-inflammatory substances, synthesized within the liver as well as carotid atherosclerotic plaques. Indeed, HCV core protein and other non-structural proteins, like NS5A and NS5B, enhance TNF-α and IL-6 levels and activates toll-like receptors associated with pro-inflammatory cytokines, suggesting that inflammation could be a mediator between HCV infection and atherosclerosis [59].

It is well known that HCV induces an increase in the hepatic levels of triglycerides, cholesterol esters, and sphingolipids, leading to HCV-induced steatosis and lipotoxicity [60]. HCV-induced steatosis, in turn, is considered a risk factor for carotid atherosclerosis independently of hypercholesterolemia, smoking and hypertension [56], since it has been associated with pro-atherogenic conditions such as increased TNF-α levels and oxidative stress [61]. TNF-α and oxidative stress also appears to influence the severity of HCV chronic hepatitis and to modulate insulin resistance, another important cardiovascular risk factor in HCV patients [62]. In fact, numerous studies have shown that oxidative stress, characterized by increased serum and liver levels of oxidation products as well as reduced liver antioxidant defenses, is present in chronic hepatitis C patients to a greater degree than in other inflammatory liver diseases [10,63].

Several factors might contribute to increased inflammation and oxidative stress in HCV-infected patients. HCV replication is associated with the endoplasmic reticulum (ER), where newly synthesized proteins enter to undergo modifications and folding, mediated by molecular chaperones and folding enzymes. Therefore, the viral replication disrupts normal ER function and induces ER stress, caused by the depletion of calcium stores, thus hindering the correct protein folding [64]. Ca^2+^ is released from the ER and it is readily taken up by mitochondria, whose increased Ca^2+^ uptake is induced by HCV core proteins as well as NS5A and NS3, causing oxidation of the GSH pool. This critical change in the mitochondrial redox state inhibits complex I activity and increases ROS production [65], which in turn activates nuclear factor κB (NF-κB) and STAT-3 transcription factors through cellular tyrosine and serine/threonine kinase pathways, leading to oxidative stress [65].

Other mechanisms proposed as a possible cause of oxidative injury during HCV infection concern increased liver iron deposition. Iron is present in many parts of the body, and liver is one of the main sites of storage; thereby, higher iron levels could result in more oxidative stress in liver cells [66]. In addition, HCV-mediated oxidative stress promotes fatty acid accumulation and β-oxidation in the liver, resulting in increased ROS production. Mitochondrial fat oxidation, in turn, upregulates NF-κB transcription factor, resulting in an increased synthesis of pro-inflammatory cytokines [67].

Recently, the introduction of new direct-acting antiviral therapy for the treatment of HCV infection has dramatically increased the sustained virological response of HCV-positive patients [68]. In addition, preliminary data provided evidence of a potential positive impact of viral eradication on cardiovascular outcomes [69]. However, future studies are needed to clarify the full impact of antiviral treatment in preventing, improving or reversing HCV-related CVD.

### 3.3. Herpesviruses

#### 3.3.1. Herpes Simplex Virus

Herpesviruses (HSVs) have been involved in the development of inflammatory atherosclerotic process [70]. In fact, the chronic inflammation mediated by HSV infection is hypothesized to promote atherosclerosis and thrombosis. A recent meta-analysis revealed that an increased risk of atherosclerosis could be observed for both HSV-1 and HSV-2 infection, suggesting that HSV may play an important role in atherogenesis [71]. In particular, evidence shows that HSV enhances the uptake of ox-LDL in endothelial cells increasing the expression of LOX-1, the major ox-LDL receptor, and attracting, thus, leukocytes, with subsequent inflammatory damage [72]. Furthermore, it has been demonstrated that saturated cholesteryl esters and triacylglycerols accumulated in VSMCs infected by HSV as compared to uninfected cells [73]. Lastly, Key et al. [74] suggested that HSV could also contribute to the deposition of thrombi on atherosclerotic plaques and induce coagulant necrosis by decreasing thrombomodulin and increasing tissue factor activity. In addition, HSV-2, but not HSV-1, was associated with premature CVD [75].

#### 3.3.2. Cytomegalovirus

Lifelong persistent infection with cytomegalovirus (CMV) has also been associated with CVD. Experimental data have shown CMV ability to infect the human vascular wall, resulting in altered function of the endothelium [76]. In particular, VSMCs isolated from atherosclerotic coronary lesions have been demonstrated to harbor CMV DNA sequences and to express immediate early proteins, which binds and inhibits p53, such as IE84 [77]. The inhibition of p53, in turn, is held responsible for the enhanced VSMC proliferation and for their impaired apoptosis, either of which may contribute to restenosis. The persistent CMV infection may also lead to endothelial dysfunction and, hence, activate pro-inflammatory signaling pathways, promoting enhanced proliferation and migration of monocytes and VSMC into the intima, lipid accumulation as well as expansion of the atherosclerotic lesion.

In the various stages of CVDs, leukocytes contribute to the initiation and progression of atherosclerotic plaques [78] by producing ROS and, hence, leading to LDL oxidation (ox-LDL). The following ox-LDL uptake by macrophages is responsible for foam cell formation and accumulation, leading to the appearance of fatty streaks on the vascular wall, an early site of potential atheroma development. In fact, CMV DNA in circulating leukocytes was considered a marker of CMV-related oxidative stress in endothelial cells and associated with transplant arteriosclerosis [79]. In addition, PCR evidence of CMV DNA in leukocytes was associated with higher oxidative stress and subclinical atherosclerosis in healthy subjects. The increased oxidative stress also resulted in mitochondrial DNA damage and dysfunction.

## 4. Antioxidant Strategies in Infectious Agent-Mediated Atherosclerotic Cardiovascular Diseases

Given the potential role of infectious agent-induced oxidative stress in the pathogenesis of CVDs, some efforts have been made to identify treatment strategies specifically directed to restore the ROS/antioxidant balance in the vascular wall.

A promising approach to reduce the oxidative stress mediated by *P. gingivalis* as well as *C. pneumoniae* may be represented by natural substances or synthesis products able to inhibit ROS production and, hence, to ameliorate the endothelial function and delay the progression of atherosclerotic plaque.

Among natural substances, green tea epigallocatechin-3-gallate (0.02%) has been demonstrated to prevent the atherogenic events induced by *P. gingivalis*, as suggested by reduced mRNA levels of oxidative stress-related mediators found in the aorta of infected mice (receptor-1 for ox-LDL, NOX, and inducible nitric oxide synthase) [80]. In addition, lipoxin A4 (500 nM), an endogenously produced eicosanoid, has been proved to attenuate *P. gingivalis*-mediated LDL-oxidation in the bloodstream via intercepting neutrophil/platelet interactions [19]. Other natural substances well known for their beneficial health properties such as curcumin (1 µM), resveratrol (25 µM), and vitamin E (50 µM) have also been suggested as intriguing candidates for reducing *C. pneumoniae*-mediated oxidative stress in monocytes, macrophages, and vascular endothelial cells, respectively [41,81]. Of particular importance are the experimental studies showing the efficacy of certain synthesis products, including anti-inflammatory drugs and statins against the atherogenic effects of *C. pneumoniae*. For example, ibuprofen and diclofenac (100 µM) have shown promising results as COX-2 inhibitors limiting ROS production in *C. pneumoniae*-infected monocytes [82]. Amongst statins, for example, fluvastatin has been shown to inhibit ROS mediated-LOX-1 scavenger receptor activity in endothelial cells [83].

Another antioxidant strategy may be represented by substances able to mimic the biochemical activity of ROS detoxifying enzymes. In fact, *N*-acetyl-l-cysteine and GSH (10 mM), natural antioxidants, have been demonstrated to inhibit ROS-mediated MCP-1 production in *P. gingivalis*-infected vascular endothelial cells [84]. Sesamol (10 µg/mL), the predominant active component of sesame seed oil, has also been evidenced to inhibit *C. pneumoniae*-mediated VSMC proliferation [85].

Regarding viral infection, the targeted use of antioxidants in therapy was explored in vivo and in vitro studies, demonstrating effectiveness to reduce virus-mediated oxidative stress. HIV-1 reduces levels of plasma antioxidants, favoring the risk of progression to AIDS in HIV-infected subjects [86]. Allard et al. [87] have found that vitamin C and E supplements reduced the oxidative damage and attenuated disease severity in HIV-positive Canadian adults. Specifically, the study was conducted in 49 HIV-positive patients randomized to receive supplements of both DL-alpha-tocopherol acetate (800 IU daily) and vitamin C (1000 mg daily) for three months. After supplementation, an increased plasma concentration of vitamins and a reduction in lipid peroxidation markers was observed when compared with controls group. In addition, a reduction in viral load was noted.

An essential role in the cellular protection is due to the nuclear factor (erythroid-derived 2)-like 2 (Nrf2) that regulates antioxidant enzyme expression. Studies, in fact, have suggested that Nrf2 activation may serve as treatment for HIV-associated vascular disorder since it does protect vascular cells against oxidative stress and inflammation. Resveratrol dose-dependently increases Nrf2 activation and stimulates Nrf2-regulated gene expression in cultured primary human coronary endothelial cells. Resveratrol also reduces mitochondrial and cellular ROS production following high glucose and TNF-α exposure in an Nrf2-dependent manner [88]. In addition, Nrf2 activation by sulforaphane reduces VCAM-1 signaling in human umbilical vein endothelial cells [89].

Clinical trials have found the effects of antioxidant, anti-inflammatory, anti-fibrotic, and anti-TNF-α compounds, such as vitamins and glycyrrhizin, in various combinations are helpful in the treatment of hepatitis C infection [90]. Indeed, several studies described improved liver function, decreased viral load, and recovered liver histology, and no major side effects after testing the above-mentioned antioxidants were found. Some of them have also been suggested to be effective in patients who did not respond to treatment with interferon. Melhem et al. [91] have shown that a combined anti-oxidant treatment is well tolerated in chronic HCV patients and may have a beneficial effect on necro-inflammatory activity. The study was conducted in 50 chronic HCV patients treated orally every day with a combination of different antioxidants at the appropriate dose for 20 weeks (Glycyrrhiza, 500 mg; Schizandrae, 500 mg; Ascorbate, 2000 mg; l-Glutathione, 150 mg; Silymarin, 250 mg; Silymarin, 250 mg; d-α-tocopherol, 800 IU/day). In addition, all patients were treated intravenously twice a week with the combination of different preparations at the appropriate dosage for the first 10 weeks of the study (Glycyrrhiza, 120 mg; Ascorbate, 10,000 mg; l-Glutathione, 750 mg; B-Complex, 1 mL). The combined anti-oxidant treatment induced a normalization of liver enzymes in patients who had elevated pretreatment alanine aminotransferase (ALT) levels, a decrease in viral load, and histological improvement in treated subjects.

Antioxidant therapy targeting mitochondria has also been proposed for the treatment of CMV-related atherosclerosis [15].

Finally, since antioxidants in chronic infected patients may reduce the oxidative damage due to the viral replication and attenuate the severity of the infection, antioxidant supplementation may represent an indirect strategy to improve cardiovascular disease. However, clinical trials failed to demonstrate the efficacy of the antioxidant therapy in cardiovascular disease. Considering the discordance of the results and the complexity of the phenomenon analyzed, others studies are necessary to verify if antioxidants may really improve the outcome of CVDs.

## 5. Conclusions

Based on the evidence above described, several infectious agents are able to interact with vascular cells resulting in oxidative stress, characterized by ROS overproduction responsible for the development and progression of atherosclerotic plaque. *C. pneumoniae* has been demonstrated to upregulate multiple enzymatic systems capable of producing ROS, such as NOX and cyclooxygenase in vascular endothelial cells, NOX and cytochrome c oxidase in macrophages, and nitric oxide synthase and lipoxygenase in platelets, contributing to both early and late stages of the atherosclerotic process. As for the periodontal pathogens, *P. gingivalis* seems be markedly involved in the atherosclerotic process as compared to *A. actinomycetemcomitans*, contributing to LDL oxidation and foam cell formation. Particularly interesting is the evidence demonstrating NLRP3 inflammasome activation as a new molecular mechanism underlying *P. gingivalis*-induced oxidative stress and inflammation.

As viruses are concerned, chronic viral infection seems to have a major role in promoting ROS induction responsible for the atherosclerotic process. In fact, the continuous production of viral proteins in the organism, such as Tat and gp120 for HIV or NS5A and NS5B for HCV, promote ROS generation alongside a decreased antioxidant production, leading to the release of cytokines and, hence, to endothelial dysfunction and LDL oxidation.

In conclusion, oxidative mechanisms activated by several infectious agents during the atherosclerotic process underlying CVDs are very complex and not well-known, remaining, thus, an attractive target for research. In future perspectives, a better awareness of infectious agents as cardiovascular risk factors may be helpful to comprehensively evaluate atherosclerosis progression in clinical practice.
